# Early Evidence for Zika Virus Circulation among *Aedes aegypti* Mosquitoes, Rio de Janeiro, Brazil

**DOI:** 10.3201/eid2308.162007

**Published:** 2017-08

**Authors:** Tania Ayllón, Renata de Mendonça Campos, Patrícia Brasil, Fernanda Cristina Morone, Daniel Cardoso Portela Câmara, Guilherme Louzada Silva Meira, Egbert Tannich, Kristie Aimi Yamamoto, Marilia Sá Carvalho, Renata Saraiva Pedro, Jonas Schmidt-Chanasit, Daniel Cadar, Davis Fernandes Ferreira, Nildimar Alves Honório

**Affiliations:** Instituto Nacional de Infectologia Evandro Chagas–Fiocruz, Rio de Janeiro, Brazil (T. Ayllón, P. Brasil, R.S. Pedro);; Núcleo Operacional Sentinela de Mosquitos Vetores-Nosmove–Fiocruz, Rio de Janeiro (T. Ayllón, F.C. Morone, D.C.P. Câmara, N.A. Honório);; Universidade Federal do Rio de Janeiro, Rio de Janeiro (R.M. Campos, G.L.S. Meira, K.A. Yamamoto, D.F. Ferreira);; Instituto Oswaldo Cruz–Fiocruz, Rio de Janeiro (D.C.P. Câmara, N.A. Honório);; Bernhard Nocht Institute for Tropical Medicine, Hamburg, Germany (E. Tannich, J. Schmidt-Chanasit, D. Cadar);; Programa de Computação Científica–Fiocruz, Rio de Janeiro (M.S. Carvalho);; German Centre for Infection Research Hamburg-Luebeck-Borstel, Hamburg (J. Schmidt-Chanasit)

**Keywords:** Zika virus, lineage, Brazil, Germany, phylogeny, mosquito, Aedes aegypti, introduction, surveillance, viruses, vector-borne infections

## Abstract

During 2014–2016, we conducted mosquito-based Zika virus surveillance in Rio de Janeiro, Brazil. Results suggest that Zika virus was probably introduced into the area during May–November 2013 via multiple in-country sources. Furthermore, our results strengthen the hypothesis that Zika virus in the Americas originated in Brazil during October 2012–May 2013.

Zika virus is an emerging arthropod-borne virus that was first isolated from sentinel rhesus macaques in 1947 in Africa. Zika virus caused outbreaks of disease in the Pacific region and emerged in northeastern Brazil in March 2015, followed by Rio de Janeiro in May 2015 (*1*–*3*). The *Aedes aegypti* mosquito is considered the main vector of Zika virus in urban and suburban areas throughout the world, including Brazil, where the mosquito has been confirmed, together with *Ae. albopictus* mosquitoes, as a vector for the virus (*4*). Entomologic surveillance for arboviruses in field-trapped mosquitoes is a critical tool for identifying local natural vectors and key sites for increased transmission risks as well as for predicting arbovirus epidemics (*5*,*6*). Therefore, virus surveillance based on field-trapped mosquitoes is a vital tool for public health authorities.

We conducted a surveillance program for mosquitoborne viruses during February 2014–June 2016 in Manguinhos neighborhood in Rio de Janeiro, Brazil. We collected mosquitoes on a weekly basis by using portable backpack aspirators and transported them on dry ice to the Núcleo Operacional Sentinela de Mosquitos Vetores-Nosmove/Fiocruz in Rio de Janeiro, where they were counted and their sex and species level were determined. 

The collected mosquitoes included a total of 417 engorged female mosquitoes (406 *Ae. aegypti* and 11 *Ae. albopictus* mosquitoes), which we pooled (n = 178) and subjected to Zika virus detection using real-time RT-PCR (*7*). Two of the pools (C20 and P52) were confirmed positive for Zika virus by conventional PCR (*8*). Pool C20 comprised 2 *Ae. aegypti* mosquitoes obtained in April 2015 from a household located in the João Goulart Park in Manguinhos; sample P52 comprised 1 *Ae. aegypti* mosquito obtained in January 2016 during a mosquito-collecting activity in a junkyard located in the São Pedro slum in Manguinhos. Sanger sequencing of the amplified fragments (*8*) confirmed the presence of Zika virus in pools C20 and P52, and we subjected both pools to deep sequencing to obtain larger fragments of the genome.

We performed phylogenetic and phylogeographic analyses based on the near-complete envelope gene sequences of Zika virus from the 2 positive mosquito pools and on all available sequences for Asian genotype Zika virus strains responsible for outbreaks in the Americas. The analyses revealed that strains from pools C20 and P52 (GenBank accession nos. KY354186 and KY354187, respectively) clustered within the same strongly supported lineage, which included strains detected in Rio de Janeiro and other parts of Brazil in late 2015 and in 2016 (Technical Appendix Figure). The mosquito-derived Zika virus detected in January 2016, strain P52, subsequently formed a subclade with human-derived Zika virus strains from Rio de Janeiro detected during March–April 2016. Furthermore, some previously reported human-derived Zika virus strains from Rio de Janeiro clustered in different lineages (online Technical Appendix Figure). The time-resolved phylogeny including the 2 mosquito strains from this study suggests that Zika virus was probably introduced in Rio de Janeiro during May–November 2013. The time to most recent common ancestor of Zika virus from the Americas is estimated to be October 2012–May 2013. These results further strengthen the hypothesis that Zika virus in the Americas originated in Brazil (*9*). The different clustering pattern of sequences of the human-derived Zika virus from Rio de Janeiro suggests that multiple Zika virus lineages may be circulating in Rio de Janeiro State.

In this study, we detected Zika virus RNA in 2 pools of engorged *Ae. aegypti* mosquitoes that were collected during a mosquitoborne virus surveillance program in Rio de Janeiro. Information regarding Zika virus infection rates is lacking for female and male mosquitoes trapped in the field. However, experiments performed in the laboratory demonstrated transovarial transmission of Zika virus among *Ae. aegypti* mosquitoes and revealed a minimal filial infection rate of 1:290 (*10*). Mosquitoborne virus surveillance provides an early warning for arbovirus circulation, points out high-risk areas for virus transmission, and provides data for directing control measures. Furthermore, future surveillance-based studies should further illuminate Zika virus ecology and patterns of spatial dynamics.

In conclusion, we showed the presence of Zika virus in engorged *Ae. aegypti* mosquitoes trapped in Rio de Janeiro before the first case of autochthonous Zika virus disease was diagnosed in the city (*3*). This finding emphasizes the importance and benefit of routine entomologic surveillance programs to public health in terms of ensuring timely implementation of disease prevention and control measures. Furthermore, considering that the analyzed Zika virus from Rio de Janeiro clustered in different lineages, our phylogenic analysis suggests multiple introductions of Zika virus from other regions of Brazil, rather than from outside the country, and an early presence (2013) of Zika virus in Rio de Janeiro State.

Technical Appendix Bayesian maximum clade credibility tree representing the time-scale phylogeny of the Zika virus outbreaks in the Americas.
