# Supplementary material for: Early Evidence for Zika Virus Circulation among Aedes aegypti Mosquitoes, Rio de Janeiro, Brazil
Source: Emerg Infect Dis. 2017 Aug;23(8):1411–2. doi: 10.3201/eid2308.162007 (PMC5547780; doi:10.3201/eid2308.162007)
Supplement: Technical Appendix — Bayesian maximum clade credibility tree representing the time-scale phylogeny of the Zika virus outbreaks in the Americas. [file 16-2007-Techapp-s1.pdf]

Article DOI: https://doi.org/10.3201/eid2308.162007

Early Evidence for Zika Virus Circulation among *Aedes aegypti* Mosquitoes, Rio de Janeiro, Brazil

Technical Appendix

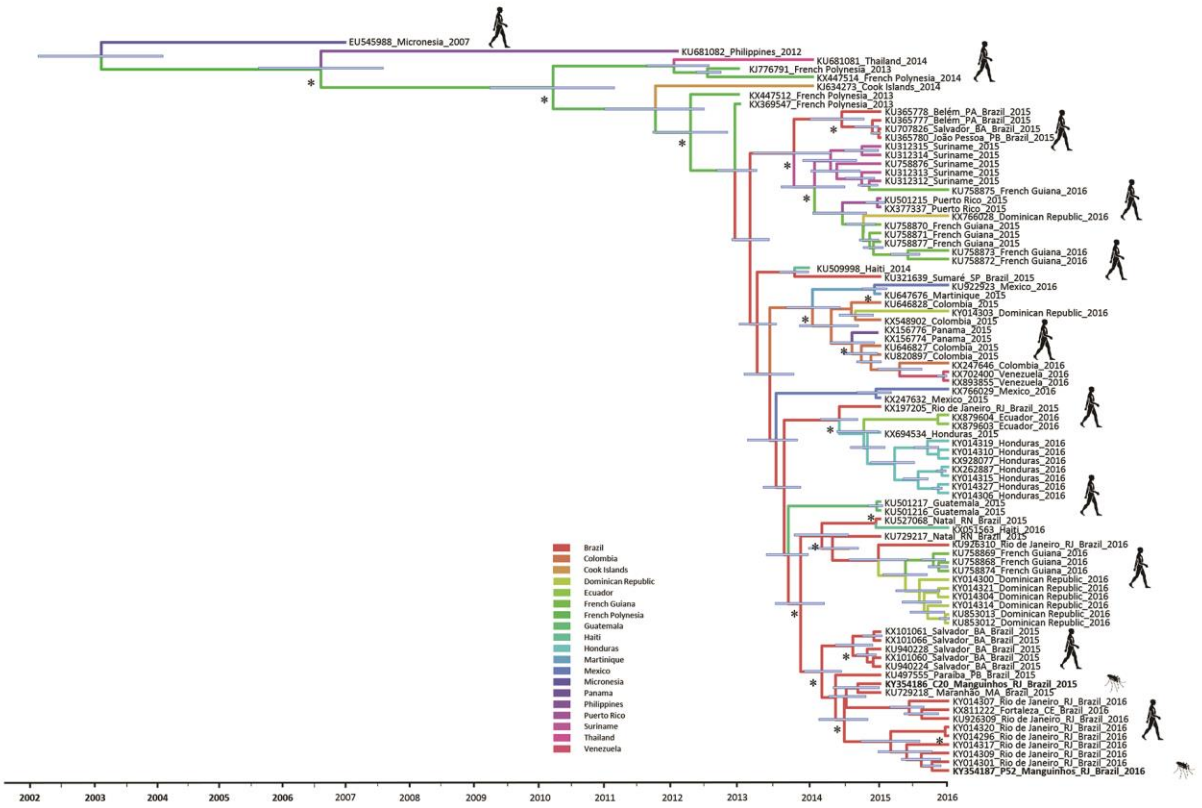

Technical Appendix Figure. Bayesian maximum clade credibility (MCC) tree representing the time-scale phylogeny of the Zika virus outbreaks in the Americas. The time-scaled phylogeny was performed by using the Bayesian Markov chain Monte Carlo tree-sampling method with BEAST version 1.8.3 (http://beast.bio.ed.ac.uk/) and in parallel the maximum-likelihood method (not shown) using PhyML 3.0 (http://www.atgc-montpellier.fr/phyml/versions.php) with 1,000 pseudoreplicates based on near-complete envelope coding region sequences. The Akaike information criterion was chosen as the model selection

framework, and the general time-reversible plus gamma distribution plus invariable site model was chosen as the best model. The Bayesian molecular clock phylogeny was estimated by using a strict molecular clock and Bayesian skyline coalescent prior. The posterior probability distributions were visualized by using Tracer version 1.6 (http://tree.bio.ed.ac.uk/software/tracer/). The colored branches of MCC trees represent the most probable geographic location of their descendant nodes (see figure key). Zika virus sequences generated from viruses derived from mosquitoes in this study are in red and bold. Blue horizontal bars represent 95% Bayesian credible intervals for divergence dates. Asterisks at the nodes represent posterior probability values (clade credibility>90%) and percentage bootstrap support values (>70%) based on 1,000 replicates. GenBank accession numbers, country of origin, and year of detection for sequences used to construct the tree are indicated on the branches. For Zika virus strains from Brazil, the location and the states of origin have been included. Brazil states: BA, Bahia; CE, Ceará; MA, Maranhão; PA, Pará; PB, Paraíba; RJ, Rio de Janeiro; RN, Rio Grande do Norte; SP, São Paulo.
